# Metabolic Syndrome and Survival in Glioblastoma Patients: Retrospective Cohort Study and Review of the Literature

**DOI:** 10.7759/cureus.53641

**Published:** 2024-02-05

**Authors:** Diana Lucas, Bruno Carvalho, Rui Tuna, Paulo Linhares

**Affiliations:** 1 Neurosurgery, Centro Hospitalar Universitário de São João, Porto, PRT

**Keywords:** progression-free survival, obesity, overall survival, metabolic syndrome, glioblastoma

## Abstract

Background

Several studies point to metabolic syndrome as a risk factor for the development and progression of several types of cancer. Its association with glioblastoma has yet to be determined, and only two studies investigate the impact of metabolic syndrome on the survival of glioblastoma patients, indicating a trend toward decreased survival in patients with metabolic syndrome. The aim of this study was to determine whether patients with glioblastoma and metabolic syndrome had a worse clinical outcome.

Methods

We retrospectively reviewed the clinical records of 180 patients diagnosed with glioblastoma. Metabolic syndrome was defined according to the American Heart Association, as the presence of at least three of the following criteria: diabetes, hypertension, hyperlipidemia, and obesity. We analyzed the overall survival and progression-free survival of patients with and without metabolic syndrome.

Results

Of 180 patients, 20 (11.1%) met the diagnostic criteria for metabolic syndrome. The overall survival of patients with metabolic syndrome was 19.8 months, and without metabolic syndrome was 17.7 months (p-value=0.085). The progression-free survival of patients with metabolic syndrome was 9.9 months, and without metabolic syndrome was 7.9 months (p-value=0.076).

Conclusion

Our results showed no prognostic relevance of metabolic syndrome in patients with glioblastoma, although there was a trend towards increased overall survival and progression-free survival in patients with metabolic syndrome.

## Introduction

Glioblastoma (GBM) is the most common and deadly malignant primary brain tumor. In recent years, there has been some progress in understanding the pathophysiology of GBM, but no improvements in patient outcomes [1].

Patients diagnosed with GBM and undergoing standard-of-care (surgery plus adjuvant radiotherapy and temozolomide) have a median overall survival (OS) of 14.6 months [2], and few survive more than two years (long-term survivors) [3].

Several studies point to metabolic syndrome (MetS), and every component of this syndrome individually, as a risk factor for the development and progression of several types of cancer. The mechanism by which MetS increases the risk of cancer is still a matter of debate, but it seems to be linked to insulin resistance [4-5].

The association between MetS and GBM has yet to be determined, and there are controversies about the impact of cardiovascular risk factors (hypertension, dyslipidemia, and obesity) and diabetes in the prognosis of patients with GBM [6], and only two studies in the literature addressed the impact of MetS in survival of these patients. According to these studies, patients with GBM and MetS had, at least, a tendency to worse OS [7-8].

The objective of this study was to determine whether patients with GBM and MetS had a worse clinical outcome. We compared progression-free survival (PFS) and OS times between patients with and without MetS.

## Materials and methods

We retrospectively reviewed the electronic clinical records of 180 patients diagnosed and treated for GBM at a Portuguese tertiary university hospital center, São João University Hospital, from 2011 to 2022.

All patients included in the study were adults, and they underwent surgical and medical treatment for GBM. Patients without any available medical information were excluded from the study.

Data collected included patient age at diagnosis, gender, comorbidities, Eastern Cooperative Oncology Group (ECOG) performance status scale, isocitrate dehydrogenase (IDH) mutation status, date of the first surgery, date of tumor progression and death, and degree of surgical resection.

According to American Heart Association (AHA) [9], MetS is defined as the presence of at least three of the following criteria: elevated waist circumference (102 cm³ in men and 88 cm³ in women); elevated triglycerides (150 mg/dL³); reduced high-density lipoprotein cholesterol levels (HDL-C) (<40 mg/dL in men and <50 mg/dL in woman); elevated blood pressure (130 mmHg³ systolic blood pressure or, 85 mmHg³ diastolic blood pressure); elevated fasting glucose (100 mg/dL³).

Waist circumference was not routinely recorded; however, all patients had body mass index (BMI) measures. BMI was used as a surrogate marker for waist circumference. Central obesity was assumed if a patient had a BMI>30 Kg/m^2^. 

Hyperlipidemia, diabetes, and hypertension were assumed if the patient was on drug treatment for these conditions. Patients with MetS had at least three of the four criteria recorded: hyperlipidemia, hypertension, obesity, and diabetes. These criteria were obtained prior to the diagnosis of GBM, eliminating the extrinsic pharmacological effect of treatment with steroids, commonly used in these patients, that can influence blood pressure, serum glucose, and weight.

All patients in this study were on Stupp protocol [concomitant administration of radiotherapy (60Gy in 1.8-2Gy fractions) and chemotherapy with temozolomide (75mg/m^2^ daily), plus six cycles of maintenance temozolomide (150-200mg/m^2^, five out of 28 days)] [10] or on a modified version of Stupp protocol with hypofractionated radiotherapy.

We considered the date of the first surgery (biopsy or surgical resection) as the date of the diagnosis. OS was determined from the date of the diagnosis to the date of death/last follow-up. PFS was determined from the date of the diagnosis to the date of radiological confirmation of tumor progression/recurrence. All patients died as a result of GBM progression.

Statistical analysis

Comparisons between patients with and without MetS were done using Chi-square and Fisher's exact tests, as appropriate, for analysis of categorical variables. Differences in the two groups in terms of OS and PFS were determined using Log-rank tests and Kaplan-Meier curves. We considered results as statistically significant at a p-value inferior or equal to 0.05.

## Results

Of 180 patients diagnosed with GBM, 11.1% (n=20 patients) met the diagnostic criteria for MetS. Eleven patients of the MetS group (6.1%) were female and nine (5%) were male. Among patients with MetS, there were 100% (n=20 patients) with hypertension, 50.0% (n=10 patients) with diabetes, 95.0% (n=19 patients) with hyperlipidemia, and 80.0% (n=16 patients) with obesity. Table 1 summarizes patient characteristics.

**Table 1:** Characteristics of patients with a diagnosis of GBM divided into groups (with and without MetS) MetS - metabolic syndrome; GBM - glioblastoma; ECOG - Eastern Cooperative Oncology Group; IDH - isocitrate dehydrogenase

Characteristics	With MetS (n=20)	Without MetS (n=160)	Total (n=180)	p-value
Sex				0.079
Female	11 (6.1%)	61 (33.9%)	72 (40%)	
Male	9 (5%)	99 (55%)	108 (60%)	
Mean age at diagnosis	63.2	58.3	58.9	0.146
ECOG				1.000
0-1	14 (7.8%)	142 (78.9%)	156 (86.7%)	
>2	1 (0.6%)	11 (6.1%)	12 (6.7%)	
Extent of resection				0.690
Biopsy	0	2 (1.1%)	2 (1.1%)	
Partial	4 (2.2%)	24 (13.3%)	28 (15.6%)	
Subtotal	4 (2.2%)	48 (26.7%)	52 (28.9%)	
Total	12 (6.7%)	86 (47.8%)	98 (54.4%)	
IDH status				1.000
Mutated	0	5 (2.8%)	4 (2.2%)	
Wild type	20 (11.1%)	156 (86.7%)	176 (97.8%)	
Hypertension				0.000
Yes	20 (11.1%)	54 (30%)	74 (41.1%)	
No	0	106 (58.9%)	106 (58.9%)	
Diabetes				0.000
Yes	10 (5.6%)	15 (8.3%)	25 (13.9%)	
No	10 (5.6%)	145 (80.6%)	155 (86.1%)	
Hyperlipidemia				0.000
Yes	19 (10.6%)	43 (23.9%)	62 (43.4%)	
No	1 (0.6%)	117 (65%)	118 (65.6%)	
BMI				0.000
<18.5 Kg/m^2^	0	1 (0.6%)	1 (0.6%)	
18.5-24.9 Kg/m^2^	0	55 (30.6%)	55 (30.6%)	
25-28.9 Kg/m^2^	4 (2.2%)	90 (50%)	94 (52.2%)	
29-30 Kg/m^2^	16 (8.9%)	14 (7.8%)	30 (16.7%)	
Progression				0.449
Yes	19 (10.6%)	156 (86.7%)	175 (97.2%)	
No	1 (0.6%)	4 (2.2%)	5 (2.5%)	
Re-operation				0.080
Yes	0	24 (13.3%)	24 (13.3%)	
No	20 (11.1%)	136 (75.6%)	156 (86.7%)	
Death				0.022
Yes	16 (8.9%)	153 (85%)	169 (93.9%)	
No	4 (2.2%)	7 (3.9%)	11 (6.1%)	

The mean age of diagnosis of GBM in the studied population was 58.9 years, and the results weren't statistically different between patients with and without MetS (63.2 vs 58.3 years, p-value=0.079).

**Figure 1:**
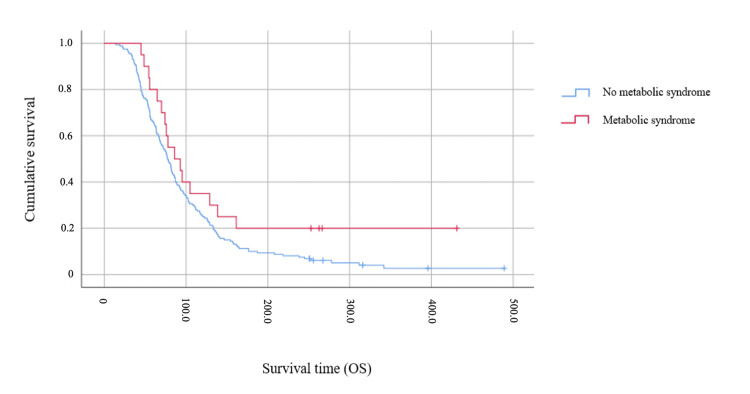
Kaplan-Meier curved for OS (in weeks) based on MetS status OS - overall survival

The median OS of the studied population was 18 months (95% CI 16.2-19.98), and the median PFS was 8.1 months (95% CI 7.1-9.2). OS of patients with MetS was 19.7 months (95% CI 12.2-27.3) and did not differ statistically from patients without MetS (17.7 months 95% CI 15.8-19.6; log-rank p=0.085), although it points to a tendency toward best OS in patients with MetS (Figure 1). The same was observed for PFS times. Patients with MetS had 9.9 months (95% CI 5.9-13.8) until progression compared with 7.9 months (95% CI 6.7-8.96) for patients without MetS (log-rank p=0.076).

We analyzed the elements of MetS individually, and we didn't find statistically significant associations between diabetes, hyperlipidemia, hypertension, and survival times (OS and PFS). Median OS for patients with diabetes was 16.6 months (95% CI 14.1-18.9) compared with 18.8 months for patients without diabetes (95% CI 17.1-20.3) (log-rank p=0.665). Median PFS for patients with diabetes was 5.6 months (95% CI = 4.6-6.7) and 8.4 months for patients without diabetes (95% CI 7.4-9.4) (log-rank p=0.572). Median OS for patients with hyperlipidemia was 16.2 (95% CI 13.6-18.9) compared with 18.8 months for patients without hyperlipidemia (95% CI 16.9-20.6) (log-rank p=0.312). Median PFS for patients with hyperlipidemia was 8.3 months (95% CI 7.2-9.5) compared with 8.0 months for patients without hyperlipidemia (95% CI 6.7-9.4) (log-rank p=0.498). Median OS for patients with hypertension was 17.1 months (95% CI 14.7-19.4) compared to 18.8 months for patients without hypertension (95% CI 17.1-20.4) (log-rank p=0.711). Median PFS for patients with hypertension was 8.3 months (95% CI 6.5-10.2) compared with 8.2 months for patients without hypertension (95% CI 6.9-9.4) (log-rank p=0.645). Obesity was the only element of MetS to show prognostic relevance. Our results showed that obese patients had a median OS greater than not obese patients (19.7 vs. 17.4 months, log-rank p=0.027). The same was obtained for PFS (11.6 vs. 7.5 months in obese and not obese patients, respectively; log-rank p=0.032). Table 2 shows OS and PFS times of the elements of MetS.

**Table 2:** Correlation between MetS components and survival MetS - metabolic syndrome

Variables	OS (months) (95% CI)	p-value	PFS (months) (95% CI)	p-value
Hypertension		0.711		0.645
Yes	17.1 (14.7-19.4)		8.3 (6.5-10.2)	
No	18.8 (17.1-20.4)		8.2 (6.9-9.4)	
Diabetes		0.665		0.572
Yes	16.6 (14.1-18.9)		5.6 (4.6-6.7)	
No	18.8 (17.1-20.3)		8.4 (7.4-9.4)	
Hyperlipidemia		0.312		0.498
Yes	16.2 (13.6-18.9)		8.3 (7.2 –9.5)	
No	18.8 (16.9-20.6)		8.0 (6.7 –9.4)	
Obesity		0.027		0.032
Yes	19.7 (15.5-24.0)		11.6 (8.7-14.6)	
No	17.4 (15.2-19.6)		7.5 (6.4-8.6)	

## Discussion

Hyperglycemia, raised blood pressure, elevated triglyceride levels, low HDL-C levels, and obesity (particularly central obesity), the set of which is known as metabolic syndrome, are risk factors for cardiovascular diseases and type 2 diabetes. The prevalence of these risk factors and MetS itself are rising, making MetS a public health problem [11-12].

Despite the high prevalence in the general population and the results of previous studies, we only found 11.1% of patients with MetS. In McManus et al.'s study, the prevalence of MetS was 18.2% (of 170 patients) [7], slightly higher than MetS prevalence in the New Zealand general population (16%) [13]. In the United States, the prevalence of MetS is 34.7% [14], and Rogers et al.'s study found a prevalence of MetS of 35.6% (of 156 patients) [8]. These studies demonstrate that the prevalence of MetS is high in patients with GBM. Within the Portuguese general population, the prevalence of MetS is also high (32.7% to 45.9%) [11-12], and according to the studies mentioned above, we were expecting a higher prevalence of MetS in our GBM patients. 

Aside from increasing the cardiovascular burden, MetS is mentioned in literature as a risk factor for the development of several types of cancer (liver, colorectal, bladder, pancreatic, breast, and endometrial cancer) [5]. The same is true for each of the components of MetS itself, existing at least a suggestion of being responsible for increasing the mortality of cancer patients [4]. The mechanism by which MetS can influence cancer risk and progression is not yet fully understood, and it may include additive and synergistic effects. Insulin resistance and inflammation are the pointed mechanisms for these associations [4-5,15]. Studies about GBM demonstrate that the insulin-like growth factor (IGF) system plays a crucial role in the pathogenesis of this tumor. Hyperinsulinemia is linked to tumor progression by activation of the IGF receptor cascade. The IGF system activates mitogenic and pro-survival mediators, contributing to an increase in GBM growth cells, cell proliferation, and migration [16-17]. 

The association between MetS and GBM has not yet been determined, and there are only two studies in the literature investigating the impact of MetS on the prognosis of these patients [7-8]. Rogers et al. demonstrate that patients with MetS and GBM who had received a full schedule of radiation and temozolomide had a median OS of 12.4 months, compared with 17.9 months in patients without MetS (p-value 0.18) [8]. McManus et al. also found a reduced OS in patients with MetS, irrespective of treatment (8 vs. 13 months, p=0.16) [7]. Our results are different from these previous studies. We did not find a statistically significant difference in OS of patients with MetS and GBM, and there was a trend toward increased survival of patients with this syndrome (19.7 vs. 17.7 months for OS, log-rank p=0.085). We also did not find an association between MetS and PFS (9.9 vs. 7.9 months for PFS log-rank p=0.076), and these results are in concordance with the findings of Rogers et al. [8]. 

We have several reasons (limitations of the study) that we think may have contributed to our contradictory results in terms of the prevalence and survival of GBM patients. In our study, we used a modification of the AHA definition. We identified patients with MetS based only on whether or not they were taking medications for hypertension, diabetes, and hyperlipidemia, and not by defined blood pressure values and serum values. We also used BMI as a surrogate for measurements of waist circumference. We think that these restrictive criteria contributed to missing some patients with MetS.

Each component of MetS has been studied in the GBM population as an individual risk factor for GBM development and as a prognostic factor, but the results are contradictory [6]. Obesity, diabetes, and hyperlipidemia are the most investigated components of MetS. Table 3 describes the results of several studies. Obesity is the only component of MetS that seems to have a positive impact on the survival of GBM patients [18-19]. We studied the impact of each component of MetS in OS and PFS of GBM patients and found that patients with BMI higher than 30 kg/m^2^ had a survival advantage of 2.3 months (log-rank p=0.027). Obese people had a median of 11.6 months before progression compared with 7.5 months in not obese patients (log-rank p=0.032). We previously demonstrated that obese patients had an increased OS compared to normal-weight patients [20]. 

**Table 3:** Revision of studies analyzing the impact of components of MetS as risk and prognostic factors MetS - metabolic syndrome

Article	Risk				Survival		
	Diabetes	Obesity	Hyperlipidemia	Hypertension	Diabetes	Obesity	Hyperlipidemia
Barami et al. [2] 2017	Null association	Null association	Null association		Reduced OS		
Rogers et al. [8] 2020							Reduced OS
Potharaju et al. [18] 2018						Increased survival BMI>25kg/m^2^	
Cha et al. [19] 2020						Increased survival BMI>23kg/m^2^	
Valente Aguiar et al. [20] 2021						Increased survival BMI>30kg/m^2^	
Seliger et al. [21] 2016	Decreased risk						
Schwartzbaum et al. [22] 2017	Decreased risk						
Disney-Hogg et al. [23] 2018	Null association	Null association	Null association				
Seliger et al. [24] 2020					Null association with OS and PFS		
Cote et al. [25] 2019			Reduced risk	Increased risk in woman			

Our results raise doubt about the protective role of MetS in the survival of GBM patients. When compared to previous studies, our results seem to suggest that obesity may, in part, be responsible for improving the prognosis of these patients. In the literature, the prognostic advantage of obese people is referred to as the "obesity paradox". Inadequate measure of adiposity by using BMI, nutritional reserves that help patients resist radiochemotherapy treatments, less aggressive forms of cancer [19], and stronger immune and inflammatory responses are possible explanations for this phenomenon [18].

## Conclusions

This study is the first to identify a trend towards increased survival of patients with MetS and GBM. In order to improve the survival of GBM patients, these studies are important to best understand the pathophysiology of this deadly tumor. Nonetheless, this study is far from reaching an understanding of the association of MetS and GBM, and more studies in this field are needed. 
